# Association between the prevalence of obstructive lung disease and the use of aspirin in a diabetic population

**Published:** 2022

**Authors:** Maria E Ramos-Nino, Charles D MacLean, Benjamin Littenberg

**Affiliations:** 1 St. George’s University, Department of Microbiology, Immunology, and Pharmacology, Grenada, West Indies; 2 Department of Pathology and Laboratory Medicine, University of Vermont 05401, Burlington, Vermont, USA; 3 Department of Medicine, University of Vermont, Burlington, Vermont 05401, USA

**Keywords:** Obstructive lung disease, Asthma, COPD, Aspirin, VDIS

## Abstract

**Background:**

Many diabetic patients take a daily low-dose of aspirin because they are two to three times more likely to suffer from heart attacks and strokes, but its role in obstructive lung diseases is less clear.

**Methods:**

A total of 1,003 subjects in community practice settings were interviewed at home. Patients self-reported their personal and clinical characteristics, including any history of obstructive lung disease (including COPD or asthma). Current medications were obtained by the direct observation of medication containers. We performed a cross-sectional analysis of the interviewed subjects to assess for a possible association between obstructive lung disease history and the use of aspirin.

**Results:**

In a multivariate logistic regression model, a history of obstructive lung disease was significantly associated with the use of aspirin even after correcting for potential confounders, including gender, low income (<USD 30,000/year), number of comorbidities, number of medications, cigarette smoking, and alcohol problems (adjusted odds ratio = 0.67, *P* = 0.03, 95% confidence interval = 0.47, 0.97). The opposite was found with aspirin and that for diabetic individuals that use insulin. A secondary analysis discovered a significant interaction between aspirin use and insulin: aspirin was associated with lower rates of lung disease except among those taking both drugs where the prevalence is significantly higher.

****Conclusion**:**

These data suggest a negative correlation between the use of aspirin and obstructive lung disease prevalence in patients with diabetes but not for those that use insulin. Further studies are required to determine if this association is causal.

## Introduction

Aspirin (acetylsalicylic acid), a non-steroidal anti-inflammatory drug, inhibits cyclooxygenases (COX-1 and COX-2), which catalyze the conversion of arachidonic acid to prostaglandins [1–3]. In control environments, low-dose aspirin (75 mg or 81 mg) inhibits COX-1 and disrupts the production of thromboxane, reducing platelet aggregation. Higher aspirin doses also inhibit COX-2, leading to the reduced production of prostacyclin (PGI2) and prostaglandin E (PGE), which is responsible for aspirin’s analgesic and antipyretic effects [4].

Several initial aspirin trials suggested that aspirin had a beneficial effect in reducing myocardial infarction and stroke, which influenced guidelines [4]. Despite these initial trials, its use for the primary prevention of cardiovascular disease remains controversial [4]. In diabetics, a few trials have shown that aspirin use failed to prevent serious vascular events without major bleeding events [5–7]. In other cases, meta-analyses and trials on men and women with diabetes have supported the view that low-dose aspirin therapy should be prescribed to patients that have suffered a heart attack or stroke to prevent future cardiovascular events [8]. New guidelines recommend aspirin use for individuals ages 40–70 at high risk of cardiovascular events and no risk of bleeding events or to individuals of all ages that have suffered a heart attack or stroke [9]. The guidelines for aspirin use keep evolving as more studies are produced.

The association between aspirin and other comorbidities has been less studied. Different phenotypes of asthma have been associated with various origins, including viruses, high T helper cell type 2 (Th2) levels, aspirin-exacerbated respiratory disease, occupation, obesity, neutrophils, and asthma-COPD overlap [10]. Aspirin-exacerbated respiratory disease is characterized by severe asthma, nonsteroidal anti-inflammatory drug hypersensitivity, nasal polyposis, and leukotriene overproduction [11,12]. The inhibition of prostaglandins by aspirin results in an excess of arachidonic acid, which then enters the lipoxygenase pathway, resulting in the increased production of leukotrienes [13]. Leukotrienes contribute to the pathophysiology of asthma, increasing airflow obstruction and the secretion of mucus [14].

Aspirin use in COPD has been associated with reduced all-cause mortality in meta-regression analyses, and daily aspirin use is associated with a reduced rate of COPD exacerbations, less dyspnea, and a better quality of life [15]. Patients with COPD demonstrate an increased platelet activation with further activation occurring during acute exacerbations [16]. Antiplatelet therapy appears to be a reasonable therapeutic choice for most patients with COPD [17,18].

Asthma and COPD have been individually and independently associated with an increased risk of type 2 diabetes, indicating that chronic airway inflammation may contribute to diabetes pathogenesis [19,20] or the other way around. In this study, we look at the association between the use of the anti-inflammatory and anti-platelet drug aspirin and obstructive lung diseases (asthma and COPD) in a diabetic cohort.

## Methods

This work is part of a larger project, the Vermont Diabetes Information System (VDIS), a study of 7,412 adults with diabetes in primary care practices [19]. The subjects comprised all diabetic adults in 64 practices in Vermont and adjacent New York. A field survey was completed to establish the study’s baseline with a subsample of subjects. Patient names were randomly sorted, and patients were contacted by telephone until a sample of approximately 15% of patients from each practice agreed to participate in the field survey to give a sample of 1,007 at the time of analysis. Four patients were dropped from the analysis due to incomplete information, leaving a final sample of 1,003.

Subjects completed a questionnaire at home and were then visited by a trained research assistant who reviewed the questionnaire responses, assisted the subject with any missing or unacceptable responses, reviewed the subject’s medications, and measured the subjects’ height and weight using a portable stadiometer and scale. Patients were asked to gather all current medications, including over-the-counter drugs. Staff recorded the drug name, dose, frequency, and route of administration from each container. Duration of therapy was not ascertained. Race, education, income, marital status, functional status, smoking, alcohol consumption, and comorbid conditions were obtained via the questionnaire. We used a modified Self-Administered Comorbidity Questionnaire [20] in which we asked each patient to indicate whether they had had the following conditions: heart attack, heart failure, peripheral arterial disease, stroke, dementia, rheumatic disease, peptic ulcer, cirrhosis, paralysis, renal insufficiency, diabetic vascular complications, AIDS/HIV, and depression. The primary outcome variable, the presence of obstructive lung disease, was the patient’s response to the question “Do you have asthma, emphysema, or chronic bronchitis?” The primary predictor variable, aspirin use, was determined by the direct observation of the container.

Most laboratory data were obtained from the patients’ local clinical laboratories, which all used the same Diabetes Control and Complications Trial/Epidemiology of Diabetes Interventions and Complications high-performance liquid chromatography (HPLC) method for the determination of glycosylated hemoglobin (A1C). Less than 1% of A1C tests were performed using the Bayer DCA 2000 immunoassay point-of-care instrument, which compares favorably to the HPLC method [21].

The research protocol was approved by the Committee on Human Research of the University of Vermont. The interviewed subjects provided written informed consent. The full study protocol and variables and the medication profiles of the subjects have been previously reported [19,22].

### Statistical approach

We used logistic regression to assess the univariate relationship between obstructive lung disease as the outcome variable and the use of aspirin as the predictor. We then adjusted for possible confounding resulting from social and clinical factors. Potential confounders tested were gender (male/female), age (years), race (white/other), body mass index (BMI) (kg/m^2^), glycosylated hemoglobin level (A1C; %), self-reported history of alcohol problems (yes/no), cigarettes (per day), low annual income (<USD 30,000 per year), duration of diabetes in years, comorbidity (excluding obstructive lung disease), insulin use (yes/no), and number of medications (excluding aspirin). To reduce the number of variables in the final model, we excluded potential confounders that were associated with the outcome in univariate analyses with P > 0.15. Such a weak association implies that the variable is unlikely to be a confounder. We used Stata/SE v.17 (StataCorp, College Station, TX, USA) for all analyses.

## Results

The study population was representative of adults with diabetes being treated in primary care practices in northern New England, USA. A total of 47.2% of this population used aspirin (Table 1).

Table 2 presents univariate associations between obstructive lung disease and the other study variables that had the potential of being significantly associated with the presence of obstructive lung disease.

Next, potential confounding variables associated with obstructive lung disease with P < 0.15 were included in a logistic regression model using obstructive lung disease prevalence as the outcome. This adjusted model showed a significant association between obstructive lung disease and the use of aspirin (odds ratio (OR) = 0.67; 95% confidence interval (CI) = 0.47, 0.97; *P* = 0.03) (Table 3).

To test the potential interaction between aspirin and insulin, a multivariate logistic model that included potential confounders was run. This adjusted model showed increased odds of having obstructive lung disease when insulin and aspirin are taken together (Table 4).

## Discussion

The American Diabetes Association indicates that for cardiovascular disease prevention, aspirin therapy (75–162 mg/day) may be considered a primary and secondary prevention strategy for those with diabetes and increased cardiovascular risk after considering the risk of bleeding [21]. In this cohort, not surprisingly, 47.2% of the diabetic patients used aspirin, giving the association between diabetes and cardiovascular disease [22]. This study found that aspirin may have benefits beyond its intended use. Taking aspirin reduces the odds of having obstructive lung disease by approximately 33% after adjusting for potential confounders.

This protection may be associated with the inhibition of thromboxane in patients with COPD [23]. Daily aspirin use is associated with a reduced rate of COPD exacerbation, less dyspnea, and better quality of life [24]. Aspirin can have an impact on inducing aspirin-exacerbated respiratory disease in asthma patients, but these patients are not prescribed aspirin and therefore did not impact the results of this study. The use of insulin was not strongly associated with obstructive lung disease in univariate analysis and was not included in the multivariate model. However, it is known that there are drug interactions between aspirin and insulin. A second logistic model confirmed the general protective effect of aspirin, except those individuals taking both aspirin and insulin had a nearly two-and-a-half-fold increase in the odds of obstructive lung disease (Table 4). Aspirin, as previously mentioned, can have a positive or negative effect on obstructive lung disease. However, insulin affects the lung by causing airway inflammation, thereby exacerbating lung disease [25], particularly asthma [26]. Aspirin stimulates insulin and glucagon secretion and increases glucose tolerance in normal and diabetic subjects [27]. In other words, aspirin enhances insulin action, which could lead to obstructive lung disease.

This study has several strengths. First, the interviewed subjects were a randomly selected subset of patients receiving primary care in the Northeast US, and the cohort is representative of primary care patients in the US. Second, the use of aspirin was determined by direct observation of medication containers. However, this study also has several limitations, including the problems associated with the self-reporting of obstructive lung disease, lack of confirmation of the obstructive lung disease diagnoses, inability to distinguish between asthma and COPD, and lack of information on the time relation between the onset of obstructive lung disease and obesity. As in any cross-sectional study, unmeasured confounders could contribute to the apparent associations found.

## Conclusion

Our findings suggest aspirin’s potential protective effect on obstructive lung disease. These results raise the possibility of using aspirin as an intervention for obstructive lung disease, but probably not when using insulin. More research is needed to identify the mechanisms at work.

## Figures and Tables

**Table 1:** Baseline characteristics of 1,003 adults with diabetes.

Characteristic	N (%) or Mean (SD)
Gender (Men)	457 (45.6%)
Age, years	64.8 (12.0)
White race	973 (97.3%)
Body mass index (BMI) kg/m^2^	33.8 (7.4)
Obese (BMI > 30 kg/m^2^)	666 (67.3%)
Glycosylated hemoglobin A1C	7.1 (1.3)
Cigarettes per day	2.8 (7.8)
Alcohol problem	78 (7.9%)
Income, median USD/year	15,000–29,999
Income below <USD 30,000	548 (59.1%)
Duration of diabetes, years	10.2 (10.3)
Obstructive lung disease prevalence	203 (20.2%)
Number of comorbidities	1.6 (1.6)
Aspirin use	473 (47.2%)
Insulin use	186 (18.5%)
Number of medications	8.3 (4.4)

SD: Standard Deviation; N: Number of subjects with the characteristic.

**Table 2:** Univariate associations between history of obstructive lung disease and other patient characteristics.

Characteristic	Obstructive lung disease patients	Other patients	Unadjusted Odds Ratio	*P*
	% or mean (SD)	% or mean (SD)		
Number of subjects	203	800		
Male, %	33.5%	48.6%	0.53	<0.01
Age, years	64.3 (11.4)	64.9 (12.1)	1.00	0.54
White race	96.1%	97.6%	0.60	0.23
Obese (BMI >30 kg/m^2^), %	77.0%	64.8%	1.82	<0.01
A1C, mg %	7.2 (1.3)	7.1 (1.3)	1.03	0.67
Cigarettes per day	4.5 (10.2)	2.3 (7.0)	1.03	<0.01
Alcohol problem, %	12.1%	6.8%	1.88	0.02
Low annual income, %	75.7%	54.8%	2.57	<0.01
Duration of diabetes, years	11.1 (10.6)	10.0 (10.3)	1.01	0.20
Number of comorbidities	2.2 (1.7)	1.3 (1.3)	1.45	<0.01
Aspirin use	39.9%	49.0%	0.69	0.02
Insulin use, %	27.2%	22.8%	1.27	0.20
Number of medications	10.2 (4.9)	7.8 (4.1)	1.13	<0.01

Each cell contains either % or mean (standard deviation)

**Table 3:** Multivariate logistic regression: obstructive lung disease vs. use of aspirin with potential confounders (N=894).

Characteristic	OR	*P*	95% CI
**Aspirin use**	**0.67**	**0.03**	**0.47, 0.97**
Gender (Male)	0.55	<0.01	0.37, 0.80
Obese	1.66	0.02	1.10, 2.50
Cigarettes per day	1.02	0.04	1.00, 1.04
Alcohol problem	1.42	0.25	0.78, 2.57
Low income	1.96	<0.01	1.30, 2.91
Number of comorbidities	1.23	<0.01	1.09, 1.38
Number of medications	1.09	<0.01	1.05, 1.14

**Table 4:** Multivariate logistic regression: obstructive lung disease vs. the interaction term use of aspirin and insulin including confounders (N=894).

Characteristic	OR	*P*	95% CI
Aspirin use	0.52	<0.01	0.34, 0.79
Insulin use	0.55	0.04	0.31, 0.96
Aspirin use * insulin use	**2.40**	**0.04**	**1.05, 5.47**
Gender (Male)	0.55	<0.01	0.37, 0.80
Obese	1.66	0.02	1.10, 2.50
Cigarettes per day	1.02	0.04	1.00, 1.04
Alcohol problem	1.42	0.25	0.78, 2.57
Low income	1.96	<0.01	1.30, 2.91
Number of comorbidities	1.23	<0.01	1.09, 1.38
Number of medications	1.09	<0.01	1.05, 1.14

## Abbreviations:

COPD: chronic Obstructive Pulmonary Disease
VDIS: Vermont Diabetes Information System
COX: Cyclooxygenase
FDA: US Food and Drug Administration
Th2: T helper cell type 2
HPLC: High-Performance Liquid Chromatography
A1C: glycosylated hemoglobin
BMI: Body Mass Index
OR: Odds Ratio
CI: Confidence Interval
SD: Standard Deviation
